# Estimating the historical minimum false‐positive risk of statistically significant reported outcomes in anaesthesia and pain medicine

**DOI:** 10.1111/anae.70142

**Published:** 2026-02-09

**Authors:** Markus Huber

**Affiliations:** ^1^ Inselspital, Bern University Hospital, University of Bern Bern Switzerland

Clinical research is driven by the search for safe and efficient interventions to improve patient outcomes. Traditionally, treatment efficiency is assessed within the framework of null hypothesis significance testing. However, this does not address a crucial question when reporting a statistically significant result: what is the risk that my discovery (in the sense of statistical significance) is wrong [1]? Equating this false‐positive risk with the p value would correspond to one of many misinterpretations of statistical testing [2]. To illustrate this problem, I will statistically estimate the minimum false‐positive risk in a large set of reported outcomes in randomised controlled trials in anaesthesia and pain medicine.

Reported outcomes were selected from the publicly available data set by van Zwet et al. [3] containing the published results of more than 68,000 studies from the Cochrane Database of Systematic Reviews. Here, only studies from the domain ‘Anaesthesia and Pain’ were selected, and only efficacy‐related outcomes from randomised controlled trials were analysed.

Two‐sided p values were computed from reported test statistics (z‐values). The false‐positive risk for each statistically significant outcome was calculated as follows [4]: $\frac{\mathrm{FPR}}{1-\mathrm{FPR}}={\mathrm{BF}}_{01}\cdot \frac{\mathrm{Pr}\left(H_{0}\right)}{\mathrm{Pr}\left(H_{1}\right)}$, where BF_01_ denotes the Bayes factor (${\mathrm{BF}}_{01}=\frac{\mathrm{Pr}\left(\text{data}|H_{0}\right)}{\mathrm{Pr}\left(\text{data}|H_{1}\right)}$). The minimum Bayes factor was calculated using the ‘‐e *P* log(*P*)’ calibration [5], which assumes p values to be distributed uniformly between 0 and 1 under the null hypothesis and smaller p values to be more likely under the alternative hypothesis.

I computed the minimum false‐positive risk for the entire range of pretest probabilities regarding the null or alternative hypothesis being true. This pretest probability could refer to the long‐term proportion of truly effective treatments evaluated in clinical trials from a frequentist perspective or the prior probability investigators assign to the efficacy of an intervention before running a trial. All computations were performed with R (version 4.4.2, R Foundation, Vienna, Austria).

**Figure 1:**
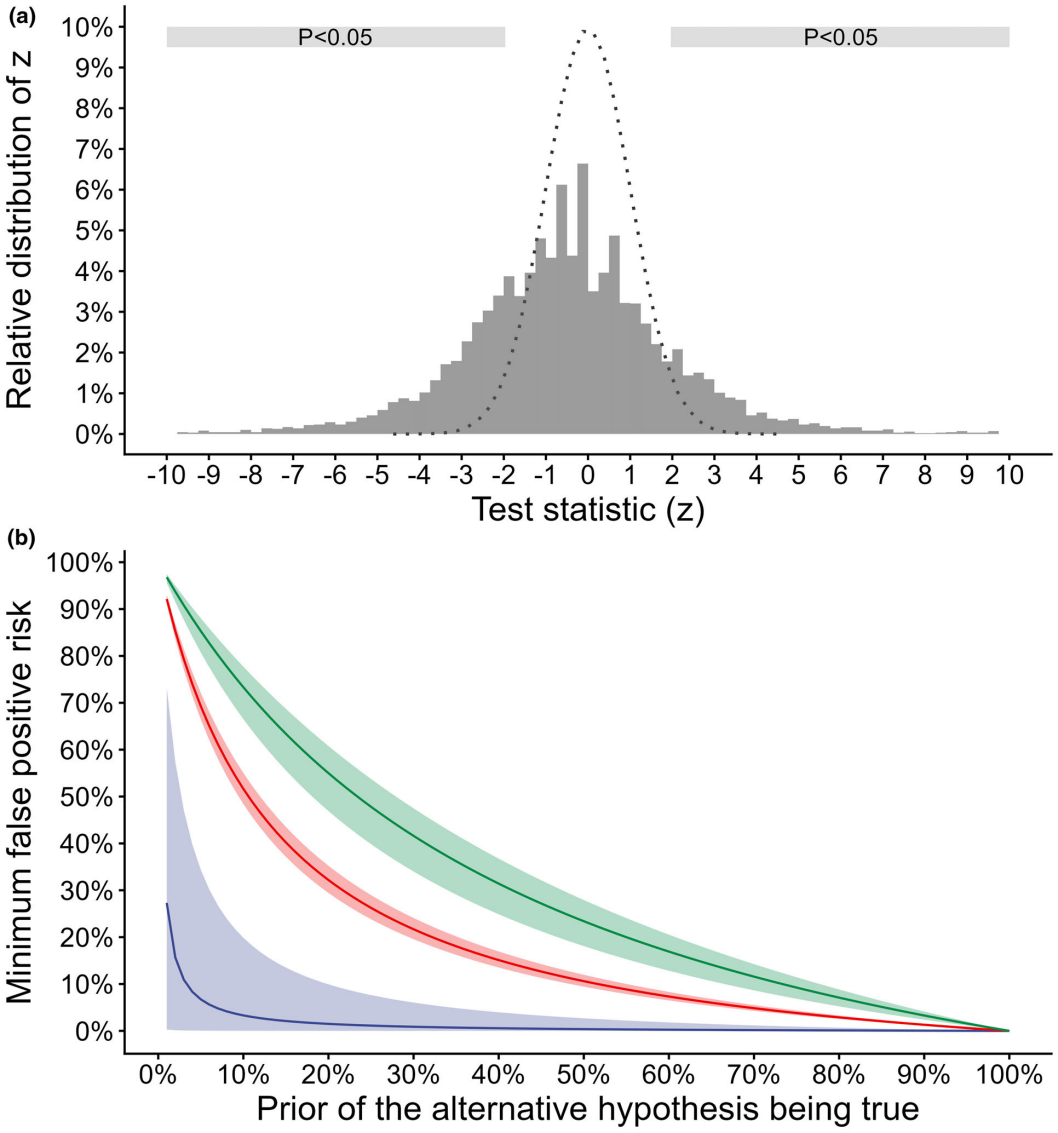
(a) Distribution of test statistics (z‐values) of randomised controlled trials in the domain of anaesthesia and pain medicine. The reported outcomes were derived from a publicly available data set [3] based on the Cochrane Database of Systematic Reviews. For reference, the standard normal distribution of z‐values under the null hypothesis is shown as a dotted line. (b) Estimate of the minimum false‐positive risk as a function of the prior probability that there is a treatment effect. The minimum false‐positive risk is stratified according to the magnitude of the outcome's p value. Solid lines denote the median minimum false‐positive risk and the corresponding IQRs are shown in shaded ribbons: blue, p < 0.005; red, 0.005 ≤ p < 0.01; green, 0.01 ≤ p < 0.05.

In total, 8707 reported outcomes from 2570 studies were analysed (Table 1). The distribution of the observed test statistics is shown in Figure 1a. The median (IQR) minimum false‐positive risk of all statistically significant outcomes was 5% (0–32%) (assuming an equal probability of the null and alternative hypothesis being true).

**Table 1:** Overview of analysed reported outcomes in randomised controlled trials in the domain of anaesthesia and pain medicine. The reported outcomes were derived from a publicly available data set [3] based on the Cochrane Database of Systematic Reviews. Values are median (IQR [range]) or number (proportion).

	Outcomes
	n = 8707
Year reported	2005 (1999–2011 [1967–2018])
z‐value (test statistic)	‐0.52 (‐1.94–0.85 [‐19.79–21.35])
Absolute z‐value (test statistic)	1.43 (0.67–2.62 [0–21.35])
p value	0.15 (0.01–0.50 [0–1.00])
Minimum Bayes factor	0.85 (0.14–1.00 [0–1.00])
Significant outcomes (p < 0.05)	3313 (38%)
Distribution of significant p values	
p < 0.005	1918 (58%)
0.005 ≤ p < 0.01	319 (10%)
0.01 ≤ p < 0.05	1076 (32%)
Minimum false‐positive risk of significant outcomes*	
All	5 (0–18 [0–32])%
p < 0.005	0 (0–3 [0–8])%
0.005 ≤ p < 0.01	11 (9–12 [8–13])%
0.01 ≤ p < 0.05	23 (18–28 [13–32])%

* Assuming an equal probability of the null or alternative hypothesis being true.

Figure 1b provides a more detailed perspective on the minimum false‐positive risk with respect to the magnitude of the p values and prior beliefs. The minimum risk of falsely declaring a treatment effect when there is none is small (e.g. < 5%) for outcomes most incompatible with the null hypothesis (p < 0.005). Figure 1b also shows that, for reported outcomes with p values between 0.005 and 0.05, a strong prior belief for the existence of an effect – or a very large proportion of truly effective interventions that are being tested in the long run – is required to reach a minimum false‐positive risk < 5%.

The importance of the false‐positive risk in interpreting the results of clinical trials is being increasingly appreciated. An analysis of the false‐positive risk in more recent trials in the journal *Anaesthesiology* showed a median (IQR) false‐positive risk of 6% (1–22%) when assuming equal pretest probabilities [6]. Similarly, the key finding of this analysis is that about half of the statistically significant reported outcomes feature a minimum false‐positive risk > 5% and about one in four outcomes feature a risk of at least 18% of rejecting the null hypothesis falsely (in the case of equal pretest probabilities).

The analysis presented here thus further strengthens previous notions that a considerable portion of findings based on statistical significance possibly declared the presence of a treatment effect when there is likely no effect [6]. As shown in Figure 1b, this is of particular importance in the case of barely statistically significant outcomes: for those 32% of the outcomes, the median minimum risk of falsely declaring the existence of a treatment effect is 23% and can reach up to 32%. These results are thus a point in case that the risk of false‐positive statistically significant findings is large when focusing on only a moderate level of significance (i.e. the commonly selected threshold of 5%) [7]. A high false‐positive risk can be further expected in the presence of underpowered studies [7]. The median power of the trials compiled by van Zwet et al. is only 13% [3] and low power is also likely an issue in the set of trials considered here. Given that reported outcomes in anaesthesia and pain medicine feature one of the largest proportions of significant treatment effects in the Cochrane Database of Systematic Reviews and larger small‐study effects compared with many other domains [8], clinicians are encouraged to consider the false‐positive risk in the interpretation of clinical trials.
